# Using Tenecteplase for Acute Ischemic Stroke: What Is the Hold Up?

**DOI:** 10.5811/westjem.2020.1.45279

**Published:** 2020-02-24

**Authors:** Tony Zitek, Ramsey Ataya, Isabel Brea

**Affiliations:** * Kendall Regional Medical Center, Department of Emergency Medicine, Miami, Florida; † Nova Southeastern University Dr. Kiran C. Patel College of Allopathic Medicine, Department of Emergency Medicine, Fort Lauderdale, Florida

## Abstract

Alteplase is the only Food and Drug Administration-approved intravenous (IV) thrombolytic medication for acute ischemic stroke. However, multiple recent studies comparing tenecteplase and alteplase suggest that tenecteplase is at least as efficacious as alteplase with regards to neurologic improvement. When given at 0.25 milligrams per kilogram (mg/kg), tenecteplase may have less bleeding complications than alteplase as well. This narrative review evaluates the literature and addresses the practical issues with regards to the use of tenecteplase versus alteplase for acute ischemic stroke, and it recommends that physicians consider tenecteplase rather than alteplase for thrombolysis of acute ischemic stroke.

## INTRODUCTION

Alteplase is currently the only FDA-approved medication for acute stroke. While alteplase has been shown to provide benefit to some patients who present with symptoms of acute stroke within 4.5 hours,^1^ its administration increases the patient’s risk of intracranial hemorrhage.^2^ Moreover, the results of some recent studies have brought up some other concerns about alteplase. One such example was the PRISMS trial.^3^ This study was terminated before reaching its enrollment goal, and was thus not definitive. However, it found that treatment with alteplase did not result in improved neurologic outcomes at 90 days as compared to aspirin for minor strokes.^3^ Additionally, a small study by Wee et al found no benefit to administering alteplase to patients undergoing endovascular thrombectomy for acute stroke due to large vessel occlusion as compared to thrombectomy alone.^4^

While not FDA-approved for acute stroke, tenecteplase has theoretical advantages over alteplase as it has greater fibrin specificity and has a longer half-life than alteplase.^5^ It is the preferred thrombolytic agent for ST-elevation myocardial infarction in the United States.^6^

Several recent studies have compared tenecteplase and alteplase with regards to their efficacy for the treatment of acute ischemic stroke. The purpose of this article is to review those studies as well as other practical matters with regards to the use of tenecteplase and alteplase. Based on these data, we make recommendations about the use of intravenous (IV) thrombolytic agents in acute ischemic stroke.

## DISCUSSION

In comparing tenecteplase and alteplase for acute ischemic stroke, there are several issues to consider: Which medication is more effective with regards to neurologic improvement after an acute ischemic stroke? Which medication has less adverse effects? Which medication is easier to administer? Which medication costs less? Each of these issues will be addressed below.

### Neurologic improvement after stroke

The results of five randomized controlled trials have been published that compare alteplase and tenecteplase for acute ischemic stroke.^7^–^11^ The first was by Haley et al, published in 2010,^7^ and it randomized patients with suspected acute ischemic stroke within 3 hours to tenecteplase 0.1 milligrams per kilogram (mg/kg), tenecteplase 0.25 mg/kg, tenecteplase 0.4 mg/kg, or standard dose alteplase (0.9 mg/kg). Patients in the tenecteplase 0.4 mg/kg group had the lowest rate of good neurologic outcomes at three months (defined as modified Rankin scale score of 0 or 1). There were no statistically significant differences among the other groups, but there was a trend towards higher percentages of patients having good neurologic outcomes in the tenecteplase 0.1 mg/kg and 0.25 mg/kg groups as compared to the alteplase group: tenecteplase 0.1 mg/kg 45.2%, tenecteplase 0.25 mg/kg 48.4%, and alteplase 41.9%

In 2012, Parsons et al published a study that randomized patients with suspected acute ischemic stroke with symptoms for 6 hours or less to tenecteplase 0.1 mg/kg, tenecteplase 0.25 mg/kg, or standard dose alteplase. A total of 25 patients were enrolled in each group. Those receiving tenecteplase had greater reperfusion on imaging studies and superior clinical neurologic outcomes at 24 hours. Those receiving tenecteplase 0.25 mg/kg had superior outcomes compared to those receiving alteplase for all efficacy outcomes, including serious disability at 90 days.^8^

Subsequently, in 2015, Huang et al published the results from a randomized trial that compared tenecteplase 0.25 mg/kg to alteplase for patients with suspected acute ischemic stroke within 4.5 hours of symptom onset. A total of 104 patients were enrolled, with 52 assigned to each group. There was no difference between groups with regards to the primary outcome of “percentage penumbra salvaged”, 68% in each group. There were also no statistically significant differences in secondary outcomes between groups, but for the tenecteplase group, there were trends towards more early neurologic improvement at 24 hours (40% vs 24%) and a higher percentage of good neurologic outcome at 90 days (28% vs 20%).^9^

In 2017, Logallo et al published the results from a block-randomized study comparing tenecteplase 0.4 mg/kg and standard dose alteplase for patients with suspected acute ischemic stroke with 4.5 hours or less of symptoms or within 4.5 hours of awakening with symptoms. A total of 549 patients were randomized to the tenecteplase group and 551 were randomized to the alteplase group. There was no difference between groups in the primary outcome of good neurologic outcome at 90 days (64% tenecteplase vs 63% alteplase).^10^

Lastly, and perhaps of most interest, in 2018, Campbell et al published the results of a study comparing tenecteplase 0.25 mg/kg to standard dose alteplase for patients with symptoms of acute ischemic stroke for less than 4.5 hours prior to thrombectomy. There were 101 patients in each group. There was a statistically significant difference between groups with regards to the primary outcome of reperfusion of greater than 50% of the involved ischemic territory or an absence of retrievable thrombus at the time of the initial angiographic assessment. This primary outcome was found in 22% of patients in the tenecteplase group as compared to 10% of those with alteplase. Patients in the tenecteplase also had superior functional neurologic outcomes at 90 days as compared to the alteplase group.^11^

Four meta-analyses have been done using the clinical trials described above.^12^–^15^ All of these meta-analyses reported no statistically significant differences with regards to neurologic recovery, and none of the meta-analyses found a difference between tenecteplase and alteplase with regards to mortality. However, the meta-analyses by Thelengana and Kheiri reported significantly improved early neurologic improvement with tenecteplase (relative risk 1.56 confidence interval [CI], 1.00–2.43; p=0.05),^13^–^14^ and the meta-analysis by Kheiri reported significantly greater complete recanalization (odds ratio [OR] 2.01; 95% CI, 1.04–3.87; p=0.04).^14^

In summary, five randomized controlled trials have found tenecteplase to be at least as effective or more effective than alteplase for neurologic improvement after acute ischemic stroke. Using the results of those five randomized controlled trials, four separate meta-analyses have been performed, and none of those concluded that alteplase is superior to tenecteplase.

### Adverse effects

All of the above clinical trials and meta-analyses measured the rates of symptomatic and total intracerebral hemorrhage.^7^–^15^ Neither of the most recent meta-analyses found a statistically significant difference in the rates of intracerebral hemorrhage between tenecteplase and alteplase,^14^–^15^ but there were trends toward less intracerebral hemorrhage with tenecteplase (OR; 0.81 95% CI, 0.56–1.17; p=0.26).^14^ Notably, this value was calculated using all doses of tenecteplase grouped together while there is evidence that the 0.4 mg/kg dose of tenecteplase might lead to higher rates of intracerebral hemorrhage compared to the preferred 0.25 mg/kg dose.^7^,^14^ Therefore, the trend towards less intracerebral hemorrhage would be more pronounced if patients who received tenecteplase at 0.4 mg/kg were excluded.

While stroke trials generally focus on intracerebral bleeding, it is worth considering the rates of other adverse bleeding events associated with the administration of alteplase and tenecteplase. There is an abundance of data from the cardiology literature comparing tenecteplase and alteplase with regards to adverse effects in patients with acute coronary syndrome. It is likely that the rates of adverse events other than intracerebral hemorrhage for tenecteplase and alteplase would be the same for patients with acute ischemic stroke as they would be for acute coronary syndrome. Thus, the relevant literature will be summarized below.

There are three randomized controlled trials that compared tenecteplase to alteplase for patients with acute coronary syndrome and reported the adverse effect of major bleeding (not just intracerebral hemorrhage).^16^–^18^ The largest of those trials was ASSENT-2, which randomly assigned patients with acute myocardial infarction to alteplase or tenecteplase. It found that patients who received tenecteplase had reduced rates of non-cerebral bleeding complications (26.43 vs 28.95%, p=0.0003) and less need for blood transfusion (4.25 vs 5.49%, p=0.0002). A meta-analysis that included that trial and the two other studies referenced above^17^–^18^ was completed, and included a total of 17,325 patients.^19^ It found a statistically significant reduction in major bleeding with tenecteplase as compared with alteplase (RR 0.79; 95% CI, 0.69–0.90; p= 0.0002). Another 2017 meta-analysis compared tenecteplase and alteplase along with streptokinase and reteplase for ST-elevation myocardial infarction.^20^ It similarly found that tenecteplase use was associated with a lower risk of bleeding than other thrombolytic regimens.

In summary, no statistically significant difference has been reported in the available literature in the rates of intracerebral hemorrhage for tenecteplase versus alteplase in acute ischemic stroke patients. Moreover, the use of tenecteplase is associated with lower rates of non-cerebral bleeding than alteplase.

### Administration

As mentioned above, tenecteplase has greater fibrin specificity and a longer half-life than alteplase.^5^ These pharmacologic differences allow tenecteplase to be administered as a bolus, rather than a bolus followed by a drip (as with alteplase). While our nursing colleagues are certainly capable of preparing and administering alteplase, the dosing regimen of 0.09 mg/kg bolus followed by 0.81 mg/kg as a drip over 60 minutes is a bit complicated. Perhaps this at least partially explains why there is data that pharmacist participation in acute ischemic stroke treatment is associated with decreased door-to-needle times.^21^ While many tertiary care facilities involve clinical pharmacists in stroke protocols, this is not feasible in rural hospitals, leaving the somewhat cumbersome task of preparing and administering alteplase entirely to nursing staff.

Additionally, the administration of the alteplase drip requires an IV pump. Not all emergency medical technicians are qualified to manage IV pumps, which may, in certain circumstances, delay or complicate a patient’s interfacility transfer. The use of tenecteplase, which does not require a pump or any specialized equipment, would simplify the administration of thrombolytics and remove one potential barrier to rapid interfacility transfer for those stroke patients who require it.

There is a great deal of emphasis on achieving rapid door-to-needle times for thrombolytics in acute ischemic stroke.^22^ However, what probably actually matters is not the door to thrombolytic initiation time, which is what is generally tracked as a quality measure, but rather the door to thrombolytic *completion time*. A patient who is given tenecteplase will have a one hour faster door to thrombolytic completion time than if they were given alteplase.

Finally, when considering the administration of tenecteplase, it should be noted that the current evidence suggests that 0.25 mg/kg (maximum 25 mg) is the optimal dose. The 0.1 mg/kg dose did not fare as well as the 0.25 mg/kg dose in the study by Parsons et al,^8^ and the 0.4 mg/kg dose may result in higher rates of intracerebral hemorrhage.^7^

### Cost

Tenecteplase is consistently less expensive compared to alteplase nationally and internationally, with one study from Nepal stating that alteplase is twice as expensive as tenecteplase ($450 for tenecteplase versus $1000 for alteplase).^23^ According to drugs.com, in the United States, a 50 mg vial of tenecteplase costs $6311.89, while a 100 mg vial of alteplase costs $9196.07.^24^ Given the doses of 0.25 mg up to 25 mg for tenecteplase and 0.9 mg/kg up to 90 mg for alteplase, it is evident that tenecteplase costs much less. Moreover, hospital prices may be much higher than those listed on drugs.com. Thus, the switch from alteplase to tenecteplase has the potential to save hospitals and patients an enormous amount of money.

### Conclusion

Tenecteplase is at least as effective as alteplase with regards to neurologic improvement after treatment of acute ischemic stroke. Additionally, tenecteplase is less expensive, easier to administer, and may have less bleeding complications than alteplase. Thus, physicians should consider using tenecteplase rather than alteplase for thrombolysis of acute ischemic stroke. If used, the preferred dose of tenecteplase is 0.25 mg/kg (maximum 25 mg).
